# Expectations and patients’ experiences of obesity prior to bariatric surgery: a qualitative study

**DOI:** 10.1136/bmjopen-2015-009389

**Published:** 2016-02-08

**Authors:** Catherine Verity Homer, Angela Mary Tod, Andrew R Thompson, Peter Allmark, Elizabeth Goyder

**Affiliations:** 1 Centre for Health and Social Care Research, Sheffield Hallam University, Sheffield, UK; 2 School of Nursing and Midwifery, University of Sheffield, Sheffield UK; 3 Clinical Psychology Unit, Department of Psychology, University of Sheffield, Sheffield, UK; 4 Section of Public Health, School of Health And Related Research, University of Sheffield, Sheffield, UK

**Keywords:** Bariatric surgery, Obesity, Shame, Stigmatisation, Patient expectation, Framework analysis

## Abstract

**Objectives:**

This study aimed to understand the experiences and expectations of people seeking bariatric surgery in England and identify implications for behavioural and self-management interventions.

**Design:**

A qualitative study using modified photovoice methods, triangulating photography with semistructured indepth interviews analysed using framework techniques.

**Setting:**

Areas served by two bariatric surgery multidisciplinary teams in the north of England.

**Participants:**

18 adults (14 women and 4 men) who accepted for bariatric surgery, and were aged between 30 and 61 years. Participants were recruited through hospital-based tier 4 bariatric surgery multidisciplinary teams.

**Results:**

The experiences of participants indicates the nature and extent of the burden of obesity. Problems included stigmatisation, shame, poor health, physical function and reliance on medications. Participants expected surgery to result in major physical and psychological improvement. They described how this expectation was rooted in their experiences of stigma and shame. These feelings were reinforced by previous unsuccessful weight loss attempts. Participants expected extreme and sometimes unrealistic levels of sustained weight loss, as well as improvements to physical and mental health. The overall desire and expectation of bariatric surgery was of ‘normality’. Participants had received previous support from clinicians and in weight management services. However, they reported that their expectations of surgery had not been reviewed by services, and expectations appeared to be unrealistic. Likewise, their experience of stigmatisation had not been addressed.

**Conclusions:**

The unrealistic expectations identified here may negatively affect postoperative outcomes. The findings indicate the importance of services addressing feelings of shame and stigmatisation, and modifying patient's expectations and goals for the postoperative period.

**Strengths and limitations of this study**

- One strength of this study is its use of a modified photovoice methodology that triangulated photographs with interview data. This combination could be applied to research with other groups where obtaining detailed in-depth evidence is challenging, for example, where it is necessary to build trust with participants who may be socially isolated or where the topic is sensitive.
- Participants were recruited from two hospital trusts based in two towns. The populations of both towns have similar significant levels of deprivation that reflects the demographics of the population accessing funded bariatric surgery, which is relatively deprived. This supports the transferability of findings to areas with comparable deprived populations.
- The study has a small sample size, however, as the aim of the study was to generate in-depth insight this was an appropriate sample size and compares with other studies of a similar nature.
- The sample contained only four men; this reflects the gender balance of the population accessing bariatric surgery services.
- This study focuses on patients’ experiences and expectations. It would be useful to expand this research to include healthcare professionals and examine their views on the patient journey, expectations and the findings regarding weight-related stigmatisation, and support required postbariatric surgery.

## Introduction

Morbid or severe obesity (body mass index (BMI) of >40 kg/m^2^) is rapidly increasing, with 2.4% of UK adults in that category.^1–2^ There is an associated health burden for patients due to obesity-related conditions including type 2 diabetes mellitus, cardiovascular disease and certain cancers.^3–5^ Severe obesity also carries an increased risk of psychological morbidity,^5^ as well as stigmatisation, intrusive reactions from others and social isolation.^6^ UK healthcare costs associated with obesity have been estimated at between £5 and £7 billion per year, a figure set to double by 2050.^7–8^ Obesity accounts for up to 7% of healthcare spending in developed countries.^8^ Bariatric surgery is a recommended cost-effective evidenced-based intervention to reduce weight and associated comorbidities in severely obese people.^4–5^ ^9–12^ Surgery is offered to patients meeting strict criteria (see box 1 for summary and National Health Service (NHS) Commissioning Board^13^). Surgery rates in England have nearly doubled, increasing from £4200 (2008/2009) to over £8000 (2012/2013).^14^ Expected outcomes from bariatric surgery include a significant and sustained reduction in weight, comorbidities and mortality, and therefore, reduced demand on healthcare services.^8^ ^13^

Box 1 **A summary of the current National Health Service eligibility criteria for bariatric surgery:**

- Body mass index of 40 kg/m^2^ or more, or between 35 kg/m^2^ and 40 kg/m^2^ or greater, in the presence of other significant diseases.
- Medical evaluation led by a formalised multidisciplinary team.
- Morbid/severe obesity has been present for at least 5 years.
- Individual complied with a non-surgical tier 3/4 service for the duration of 12–24 months.

In England, the NHS recommends that weight loss and obesity services are delivered through a tiered model,^13^ tiers 1 and 2 being universal and lifestyle intervention. Tier 3 delivers specialist obesity services by a multidisciplinary team (MDT). Tier 4 is surgically led multidisciplinary specialist services providing predominantly bariatric surgery.^15^ Delivery of this tiered model across England varies, and responsibilities for commissioning the tiers lie with different organisations including NHS England, Clinical Commissioning Groups and Local Authorities. The tiered model should ensure patients are appropriately selected for bariatric surgery and receive adequate physical, psychological and educational preparation.

Preoperative preparation should include support regarding the postoperative behaviour change that is required following bariatric surgery. Self-management may be effective in promoting behavioural change prior to and following bariatric surgery within a tiered service pathway,^16^ however, there is no research demonstrating this.

Severely obese people, who have repeatedly lost and gained weight, consider surgery to be the ‘last resort’.^17–19^ There is an indication that the desire for bariatric surgery is associated with high, even transformational, expectations of improved physical, emotional and relational well-being.^18–20^ However, some bariatric surgery patients fail to sustain weight loss, and reasons for this remain unclear.^6^ In order to maximise the attainment of positive outcomes following bariatric surgery, there is a need for research examining the effectiveness and experience of behavioural and self-management interventions.^21^ There is a requirement to better understand patients’ expectations and experiences across the service pathway. Little is known about the weight-related presurgery experiences and expectations of bariatric surgery patients who have gone through a tiered service model. This paper reports a qualitative study, using a modified photovoice approach. The study aimed to answer the following questions: first, what are the experiences and expectations of people seeking bariatric surgery in England; and second, what are the implications of the findings for behavioural and self-management interventions. The aim was to provide an account of patient experiences elicited shortly before bariatric surgery. The intention is to generate insight and understanding to help inform the commissioning and delivery of weight management services that provide the required preparation for patients prior to bariatric surgery.

## Design

This prospective qualitative study used a modified photovoice methodology incorporating photography, semistructured individual interviews and framework analysis techniques.^22–24^ Photovoice is a participative research approach traditionally used in a community context where participants take photographs to illustrate their experiences of the issue of concern and the meanings they hold for participants.^22^ In this study, the focus of concern was obesity and bariatric surgery. Photographs were taken by individual patients, rather than community members, and used to guide the semistructured individual interviews. Framework analysis was used as it enables the use of a priori knowledge in the development and refinement of the thematic framework. An inductive approach was used, where emerging data was used to develop, refine and verify themes and findings.

## Setting

The study was conducted in areas served by two hospital-based, bariatric surgery, MDTs in the north of England. Data were collected between August 2012 and April 2013.

The study was conducted prior to the NHS England commissioning guidance published in April 2014.^13^ This guidance sets out eligibility criteria for the commissioning and delivery of NHS-funded morbid obesity surgery, stating the requirement for all patients to have accessed tier 3 support prior to referral for tier 4 surgery. The two hospitals received referrals from areas which differed in the routes to bariatric surgery. Participants in this study were recruited from three areas which referred patients through the recommended community tier 3 services, and one area which referred via the primary care physician.

## Sample

Eighteen participants were recruited prior to bariatric surgery through two bariatric surgery MDTs. Sixteen had been to tier 3 community obesity services with access to dieticians, obesity nurses, talking therapists and general practitioners (GP). Three others were referred directly from a primary care physician, as their area did not have a tier 3 service. All participants were over 18 years of age, having surgery for the first time and undergoing a gastric bypass, gastric band or gastric sleeve. Purposive sampling^23^ ^25^ was used to select participants to ensure that the sample had the necessary variety of characteristics in terms of age, gender, employment comorbidities and marital status. The study team did not have access to medical records, so comorbidities and BMI were self-reported by some of the participants. During the study period, we were only able to recruit four male patients, however, this is reflective of the proportion of men within the surgical population. The characteristics of the sample are summarised in table 1.

**Table 1:** Sample characteristics

Participant	Age (years)	Gender	Marital Status	Comorbidities	Employment Status	Referral route
1	35	F	Divorced	Joint pain	Unemployed	Tier 3 weight management service
2	54	F	Married	Type 2 diabetes, cardiovascular disease, depression	Unemployed	Tier 3 weight management service
3	46	F	Divorced	Depression, osteoporosis, asthma, hypermobility syndrome,	Unemployed	Tier 3 weight management service
4	61	M	Married	Type 2 diabetes, cardiovascular disease, psoriasis, thyroid disease, obstructive sleep apnoea	Retired	Tier 3 weight management service
5	57	F	Divorced	Joint pain, depression, type 2 diabetes, obstructive sleep apnoea	Full time Employed	Tier 3 weight management service
6	36	F	Married	Joint pain	Employed	Tier 3 weight management service
7	53	F	Married	Fibrinolytic defect, cardiovascular disease, joint pain, history of depression	Retired—ill health	Tier 3 weight management service
8	34	F	Cohabiting	Type 2 diabetes	Unemployed	Tier 3 weight management service
9	61	F	Cohabiting	Type 2 diabetes, joint pain, acid reflux, cardiovascular disease	Retired	Diabetic consultant
10	59	M	Single	Type 2 diabetes, obstructive sleep apnoea	Full time employed	GP
11	53	F	Married	Joint pain, type 2 diabetes, fibromyalgia, Crohn's disease	Retired—ill health	Tier 3 weight management service
12	33	F	Married	None reported	Unemployed	Tier 3 weight management service
13	30	F	Married	Depression, asthma	Unemployed	Tier 3 weight management service
14	50	M	Single	Cardiovascular disease, mental health condition	Unemployed	Diabetic consultant and tier 3 weight management service
15	48	M	Married	Type 2 diabetes, history of depression	Full time employed	Diabetic consultant and tier 3 weight management service
16	49	F	Cohabiting	Joint pain, history of depression	Full time Employed	Tier 3 weight management service
17	30	F	Married	Polycystic ovary syndrome, diverticulitis	Full time employed	Tier 3 weight management service
18	52	F	Married	Joint pain	Full time employed	Tier 3 weight management service

GP, general practitioners.

## Study procedure

Participants were recruited through hospital-based, bariatric surgery, MDT. The photovoice tasks and interview schedule were developed through consultation with the MDTs, patient and public involvement with previous bariatric surgery patients, and relevant literature. Photovoice tasks or ‘assignments’ were given to participants prior to the interviews. The assignments included safety instructions for taking photographs and explained what would happen to the photographs. Participants were given prompts on what the photographs could include. These were to explore life as an obese person, the decision to be referred for the surgery, preparation for surgery and expectations of how life will change after the surgery. The resultant photographs were used as prompts in the interview. The study received NHS Research Governance approval. Ethical approval was obtained from Leeds East NHS Research Ethics Committee. The study had independent scientific review through Collaborative Leadership in Applied Health Research and Care—South Yorkshire (CLAHRC-SY), and a patient and public involvement group with CLAHRC-SY.

## Data collection and analysis

At the time of interview none of the participants had a confirmed date for their surgical procedure, they were either waiting to see or had recently met their surgeon for the first time. Seventeen of the 18 interviews were carried out in the participants’ homes and 1 in the principal investigator's office in the research centre. Interviews started by asking the interviewee to show the researcher their photographs and explain why they took them and what they meant. An interview guide was referred to throughout. This guide was developed by the research team, with reference to the research questions and aims, and informed by relevant literature and policy.^24^ The photographs were discussed first to ensure that the participant's experiences led the data collection. Any topics not covered by the photographs were asked at the end. The interview schedule explored history of weight, decision to have surgery, expectations of the surgery, and type of support received when interviews were recorded, transcribed and checked before being entered onto NVivo V.10. Semistructured interviews ranged between 32 and 104 min. Participation in the photovoice methodology was left to the discretion of the participants. Fifteen participants took part in the photovoice tasks, the other interviews were guided purely by the interview schedules. Photograph data were also entered into NVivo.

The intention was to stop data collection and the recruitment of new participants once no new themes were emerging from the analysis, and data saturation was said to have been reached.^26^ This was at 15 participants. However, 18 participants were finally recruited to ensure we had a sufficient sample if anyone decided to withdraw from study.

Data were analysed using framework analysis.^23^ Framework analysis involves a systematic process of sifting, charting and sorting the material into key issues and themes allowing the integration of pre-existing themes into the emerging data analysis. The photographs were used alongside the interview transcripts in the familiarisation stage to generate an initial thematic framework. Knowledge from existing evidence and policy was also integrated into the initial thematic framework.

The interview transcripts were then coded to test, expand and verify the initial thematic framework. In this way, previous evidence and preconceptions were challenged. As a result, themes were added, removed and merged following discussion with the project team. The photographs were used alongside the transcripts to check, challenge and confirm the ongoing interpretation in an inductive way. Using the photographs alongside the transcript data added to the depth of insight, and enabled tangible verification of the interpretation of the written data. Analysis was led by the principle investigator (CVH). AMT audited the analysis process by reading seven of the transcripts alongside the photographs to verify the themes. The other authors (AMT and PA) reviewed the transcripts and photographs. They contributed to the analysis and final results by providing additional interpretation.

## Results

**Figure 1:**
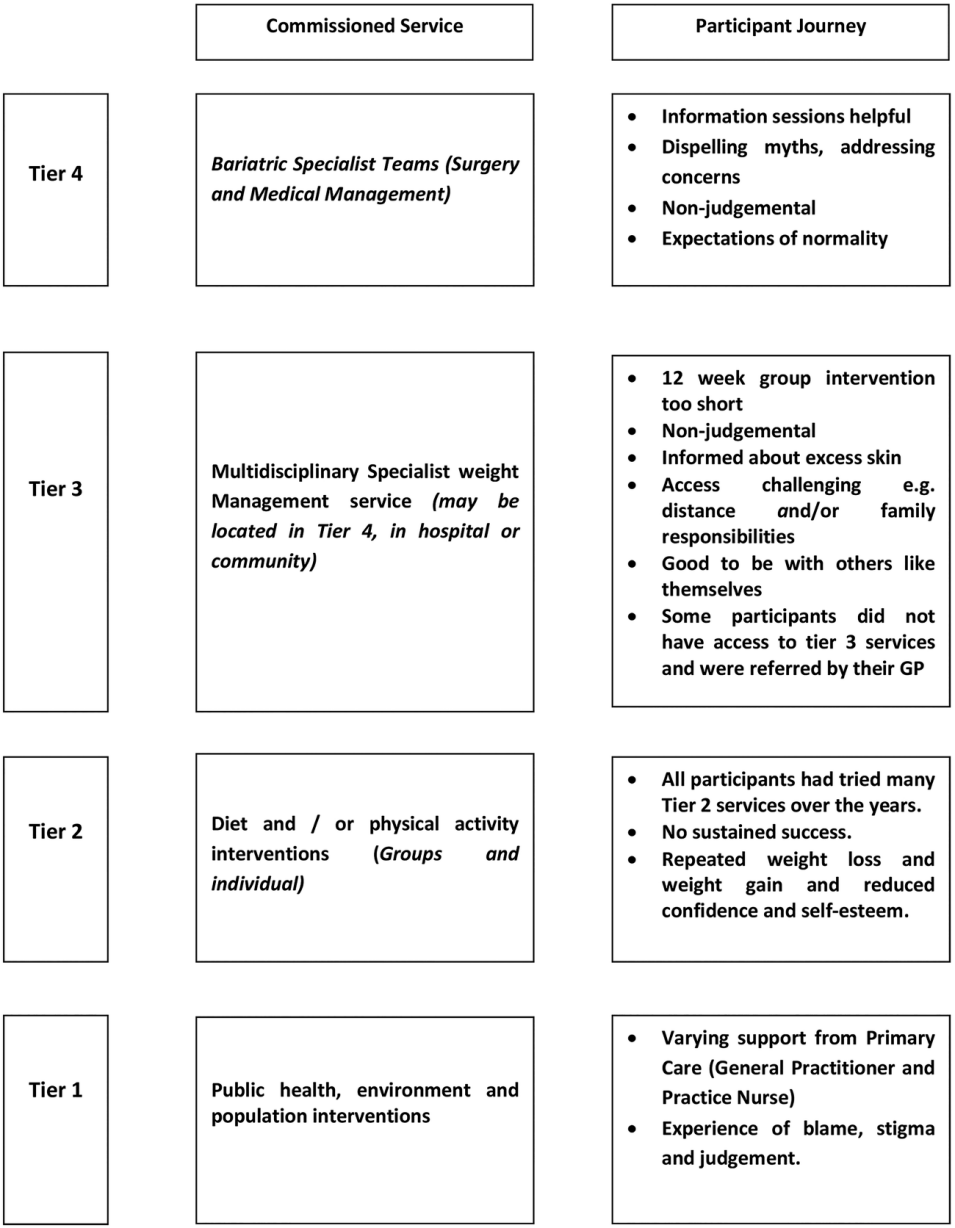
Tiered service model.

The findings are reported under three broad headings: the negative experience of obesity, experience of weight management services, and expectations of normality. Quotes and individual participant's experiences are provided to illustrate the findings (boxes 2–4). Figure 1 displays the services participants accessed at each tier and some of the key themes that were evident at each stage.

Box 2 **Negative experiences as an obese person of self-blame, shame and stigmatisation**

“Let's say I've got to do it [bariatric surgery]

because I know that I'd be dead if I didn't…..it's [obesity]

affecting my life in that I can't run around, I can't walk far. I've got to do something about it, I realise that, because I know I'll be in a wooden box if I don't do anything about it." (P10)

“You're fat, it's your own fault, do something about it, get on with your life. But when you feel that low it's not easy. See and then you don't talk about it because it makes you cry and then you feel like a silly cow because you're crying. And it's only because you know it's your weight and it's your own fault, and that's what I do, I blame myself all the time. And it is my fault and I know it's my fault, and I hate crying because it makes me look so weak and pathetic” (P5)

"I would like somebody to walk in my shoes every day and see what I have to put up with, the gestures that you get off people saying oh fat this, fat that.” (P6)

"I don't go on picture. I'm always the one to take the pictures. Do you know what I mean, I get out of it that way—oh I'll take them! Because if I look at myself, in my mind I'm saying to myself oh my God, I need to get shot of that, I need to burn it. I've found loads of photographs and burnt them. Put them in fire, ripped them up, put them in fire, and if we've had fire out then put them in a bag” (P7)

"I get it into my mind I'm going shopping, food shopping for the house. I go out, do it and come home. It's done, that's a job done. I've got to go to hospital and have this, this and this done, home. On the way there I'm thinking how long am I going to be there before I get home? My home's my lifeline, it's my haven; it's where I hide.” (P7)

"You see mums talking in the playground and they're all socialising, but I don't know anybody, I drop my kids off and I come straight back out. I don't talk to anybody” (P12)

“That's sort of the window in the door, meaning like to go outside, and the blinds are closed because then I can't see the outside and it can't see me…..It's a protection thing, it's complete protection. If I don't have to go outside into the outside world then I'm safe in here. This is my safe place.” (P1)

Box 3 **Experiences of tiered obesity services**

Participant explaining previous experiences of trying to access surgery before the tiered system:

 “I went to see my doctor because I got, you know, depression with the size that I am, and she just happened to say have you considered a gastric band, which I'd been trying for the last three, four year, and I just got pushed from one department to another" (P2)

The tier 3 service:

“It's not long enough. It's not long enough. People who've got a smoking or a drinking problem or a drug problem get longer than that, and you know, and weight is an issue. And it is an illness" (P11)

"I did get involved with that, [exercise groups at tier three]

but the only problem is I've got to go there, and a lot of the time I can't get out because of my ankles and my legs swell up…… so I have to get taxis which is very difficult. I've got limited income as well, so that makes it very difficult as well" (P14)

”the problem with the [tier 3 service] is, because they do groups and they do sort of weighing sessions, but they're all when I'm working, so it's absolutely useless for me now. Unless I have made an appointment it doesn't work. And even the last appointment that was available was I had to go to work early to get in to finish to get there, so it was difficult to access everything all the time, because it wasn't flexible for working people" (P17)

"I wouldn't go to gym because you'd feel stupid because I did try it after but I thought I can't, I was like having panic attacks and I thought I've got to get out of here. But going to that it really helped. If there was somewhere like that I could go to on a regular basis I'd love to do that” “**Do they not continue then that after your 12 weeks?"** “No it finishes then” “**You can't keep going?"** “No it finishes" (P5)

"That [hospital seminar] were brilliant. If I'd had that information before, I'd have known exactly what I were going to go for. I went in thinking right, I'm having gastric, I'm going to go for the gastric band, I come out thinking right, I've put my name down for a gastric sleeve, which is completely opposite” (P2)

**"**they [tier 3 service] are big on checking that everyone's ready for what they're doing, and they won't even put you forward if they don't think you're ready….because you have to show commitment if you don't show that you're committed to doing what they're asking you to do they're not going to refer you for the surgery" (P13)

Box 4 **Expectations of normality following surgery**

"I don't want to be slim, I want to be normal, I want to be healthy and that's all I want to be. I don't want no miracles". (P5)

“The diabetes will go, hopefully, the apnoea will go, hopefully, a lot of these things will correct themselves so that will have a big, big effect on my life." (P10)

"But with this bariatric bypass then it's supposed to get rid of like most of the diabetes cases. So I'm hoping to do away with all that medication, which it's a pain every morning. I'm 61, I get forgetful, sometimes I forget to take my tablets, if I get up feeling great, and then it'll dawn on me when I start to feel terrible later on in the day, I think oh no I've not had my tablets. And so like you're dashing about having these tablets and injections, and then it throws your routine out and it's a bind, it is a bind.” (P11)

"I can go back to doctor's well look I'm skinny, I've still got this problem what are you going to do about it? Because something they always relate back to is it's because of your weight. So if the weight's not a problem what else can they do?” (P17)

"If you're just on a diet you think oh we're going to go for a meal tomorrow, oh I'll have a day off. But once you've had that surgery there isn't any having days off is there” (P15)

"I need to figure out how I'm going to change it to incorporate these social events. But like again my friend's sister she drinks like a trooper and she's had it done. So it's not that she can't ever drink again, it's just that there's a limited time that you can” (P17)

“I'm so excited about this bariatric treatment because I'm going to get into that dress, and I will get into it” (P9)

"It's to do with just normal things and confident to be able to go to Alton Towers and confident to walk into a shop and know that something's going to fit me or that sort of confidence. And confidence as well that I can lose weight and continue to do it, because it's something that I've never been able to do.” (P17)

## Negative experiences of obesity

The combination of photographic and interview data revealed how profound the impact of obesity was on the participant’s emotional well-being and quality of life (see box 2). For some people, this was so marked that they described their life as not worth living: ‘I don't care anymore, just get me out of this world, I've had enough’ (P7). The impact was compounded by years of weight cycling through attempts at weight loss techniques, diets and exercise regimes. Surgery was considered by all participants to be the last resort. Over half the participants said that without surgery they may as well be dead or would not have long left to live.

Participants who suffered from weight-related comorbidities that required multiple medications found this polypharmacy burdensome and constricting, and also impaired their quality of life.

Employed participants described work as having a positive effect on self-esteem. Work and the interaction with colleagues gave them a purpose in their life, and an identity other than just being a ‘fat person’. However, unemployed participants described how their weight and associated poor health prevented them applying for, or staying at work, further reducing their self-esteem.

Nearly all participants reported feeling stigmatised because of their weight, and some had received negative and judgemental comments from strangers. However, such negative responses were not just received from strangers. Participants described how family members did not understand their position and appeared to judge or blame them for their obesity and related health problems. Prior to being referred to specialist obesity services, participants identified that healthcare professionals had also been judgemental regarding their weight. Participants reported how their families did not understand their weight struggles, and viewed the surgery as an easy or soft option. This lack of understanding from the healthcare profession and of those closest to them meant that participants felt increasingly marginalised from networks they regarded as their support.

Participants described long-standing shame and embarrassment regarding their appearance, day-to-day activities, and health. Participants were self-critical, and many had reached a stage where they avoided social situations, family and friends. They felt ashamed of their appearance and were worried that others would think they had ‘let themselves go’. Activities normal to others, for example, trips and holidays often caused anxiety. Female participants with children found getting their children ready for school and the journey to school challenging. It was worsened by a fear of being talked about by other parents because of their weight. As a result, they avoided situations that required speaking to other parents. Participants described being a burden to others in the family and blamed themselves for restricting their family's social lives, opportunities for holidays and enjoyment of life, all worsening their self-esteem.

Navigating space both inside and outside the home presented daily functional challenges for the participants. This meant they were unable to live what they considered to be a ‘normal life’. Even attempts to lose weight were hampered by feelings of not belonging in places where ‘thin people go’, for example, gyms. Home became a haven and was seen as a safe space offering protection from the outside world, and five of the participants only left the house when absolutely necessary.

Excess weight created practical problems and was reported as being associated with pain, comorbidity and immobility; these gradually reduced their ability to perform daily tasks, such as climbing stairs, cooking, cleaning and personal hygiene. These practical difficulties had reinforced the negative psychological impact.

## Experience of weight management services

Participants’ experiences of tiered weight management services are illustrated by the quotes in box 3. Prior to referral and, in particular, the development of the tiered service framework, few participants realised that NHS-funded bariatric surgery could be available to them. In desperation, they had tried to access privately funded procedures. Participants reported problems when negotiating clinical pathways to access the right departments for weight management. Once patients had realised that funded surgery was a possibility, they became fixed on the notion of it as the only solution to their obesity.

Tier 3 weight management services operated differently across the geographical areas of this study, largely due to changing commissioning policy and service specifications. Participants who had access to, and attended a 12-week weight management service felt this was not long enough. One participant proposed that treatment for obesity should be like that for addictions and not time limited. All participants who attended tier 3 weight management services commented that they were delivered in a non-judgemental way. This compared favourably with participants’ contact with other health professionals in other settings where they recalled feeling blamed because of their obesity and related health conditions.

Participants hampered by mobility problems and low self-esteem found it challenging to attend weight management sessions that were held in central locations and required long journeys. Employed participants referred to the struggle for them or family members to fit appointments around work. Those that attended exercise sessions praised the impact they had on mobility and health. They also reported feeling comfortable as they were exercising with ‘similar’ people. However, they were often deterred from exercise at the end of the 12 weeks when they were encouraged to attend public sessions with people who were not obese. Cost was also a consideration for many; exercise classes were free or at a reduced rate in the first 12 weeks, but some felt they could not afford to attend at unsubsidised cost.

All participants had tried dieting in the past, and despite widely available information about portion sizes and healthy eating, participants reported the benefit of receiving specific advice from specialist weight management dieticians. The personalised information rather than a generic ‘diet sheet’, and the opportunity to revisit concerns about diet were deemed to be helpful in maintaining improved eating habits.

There was variation in the level of knowledge about bariatric surgery among participants. Some had friends who had already had surgery; for others, it was their GP or tier 3 service staff who first mentioned surgery. Participants did describe that staff at tier 3 attempted to prepare participants for what life would be like after surgery, and trusted websites were given for patients to undertake their own additional research. However, during the transition between tier 3 community weight services and tier 4 specialist bariatric services, patients felt unsure where to access support. Once referred for surgery, participants attended an information seminar at a hospital. In the cases of the two participants who had not had any additional weight loss support, this was one of the first occasions they had realised there were ‘others like me’, or were given any information about the surgery. There were contrasting experiences of the seminars. Some thought that the seminars were informative, and dispelled myths and concerns the participants had while helping them to decide which type of surgery best suited them. Others commented that the seminars were basic and offered no new information they could not have found on the internet.

Generally, participants were surprised at the speed of the process between primary care and weight management services and the bariatric surgery team. However, once referred for bariatric surgery, comorbidities, such as obstructive sleep apnoea needed to be controlled prior to surgery; this lengthened the referral process and frustrated participants. At all stages of the surgery pathway, there is an expectation that patients demonstrate commitment to changing and maintaining their eating and exercise behaviours in order to be listed for surgery. Many participants in this study emphasised their fear of being refused what they perceived to be life-changing surgery if they did not change their behaviour or manage comorbidities. They were determined to show the commitment required.

## Expectations of normality

Unrealistic expectations of surgery were reported by all participants (see box 4). There was an expectation of improved health, and an eradication or reduction in comorbidities. They looked forward to a time after surgery, when burdensome medication for weight-related comorbidities would not be required. Participants reported feeling blamed and stigmatised by health professionals as a result f their weight. They anticipated that the weight loss following bariatric surgery would lead to a improved relationship with health professionals.

All participants acknowledged that changes to diet and physical activity were essential if the surgery was to be successful in the long term. However, people varied in terms of the extent to which they described a commitment to change behaviour. Surgery was commonly referred to as a ‘tool’ to control eating, rather than participants needing to take responsibility for their eating behaviour. While some recognised that personal control would still be required, others had unrealistic expectations that surgery would remove the need for their decision to eat or not. Half the participants knew others who had had surgery, and used their experiences and success as a benchmark for the extent of behaviour change required. Unrealistic hopes that they could retain some current behaviour and still lose weight after surgery were derived from the personal stories of other people.

Female participants had taken photographs of clothes and underwear against furniture to indicate ‘how big’ they were. Contrastingly, other photographs of smaller sizes of clothes in shops demonstrated the hopes that came with the surgery. While participants were extremely optimistic about the anticipated physical changes, they also raised concerns about the reactions of close family and friends provoked by changes to image and identity. This was particularly apparent in those who reported they ‘had always been big’. Social isolation was anticipated to reduce as many hoped changes in their weight would mean they would have more confidence to go out without worrying what strangers thought about them.

All participants were aware of potential problems concerning excess skin, but did not believe this would be an extensive or distressing issue for them. They anticipated that the improved changes to appearance from losing weight would, by far, overcome any concerns they had about excess skin. Older (50 years plus) female participants joked how they would ‘just tuck it in’.

Participants had great hopes and expectations regarding increases in confidence, motivation and overall zest for life following their surgery. They reasoned that weight loss and improved mobility and health would remove their life and emotional challenges, and help them to feel like a ‘normal’ person again. Participants anticipated that the weight loss following surgery meant they would no longer be viewed as ‘different’. They expected their confidence to increase to an extent they would be able to manage any negative comments and stressful situations, even with small weight loss.

## Discussion

This research responds to the call for more evidence to increase understanding of bariatric surgery patient experience.^27^ The study provides new insight from the perspective of the participants, into the period prior to bariatric surgery in England. The findings indicate the extent of obesity-related distress experienced in life prior to bariatric surgery. Desperation for surgery, and extensive expectations of life after surgery, were evident. This study supports previous findings in terms of the extent of bariatric surgery patients’ psychological and physical morbidity.^18–20^ ^28^ However; this study adds new information about how the impacts of obesity play out in everyday lives, creating low self-esteem, social avoidance and poor quality of life prior to different types of bariatric surgery. Taking refuge at home increased social isolation and intensified feelings of worthlessness. Such preoperative experiences were seen, in this study, to exacerbate unrealistic aspirations for postoperative normality.

There is growing evidence that patients face problems because of excess skin postsurgery.^29^ Our participants reported similar problems. What is new in this study is the finding that, despite being informed by the MDTs of the possible consequences, the majority of the participants in this study rejected the notion that excess skin would be a problem for them. While the participants knew about excess skin prior to surgery, they thought it may be a problem for others but wouldn't be so for them. They did not anticipate that excess skin would obstruct their journey to ‘normality’.

Previous evidence has focussed on the weight loss goals of patients. This study provides new insight of participants’ broader expectations of ‘normality’ regarding weight and appearance, eating and activity behaviour, social life and emotional resilience following surgery. Questions emerge regarding how feasible these expectations of normality are and, if unrealistic, how this could impact on the success of surgery outcomes.

The social and emotional burdens of obesity were reported as major factors to patients accessing bariatric surgery services. Participants reported negative reactions from others in the past regarding their obesity, which often led to social avoidance. The tiered service framework provided access to support and information in preparation for surgery. However, unrealistic expectations of surgery had not been detected, challenged or modified. The hope and belief that life following bariatric surgery would become ‘normal’ was evident across all interviews, but there were differences in the extent to which people indicated an ability or willingness to embark on behaviour change and self-management strategies themselves. While some participants saw surgery as a trigger for change, others saw it as a tool that meant little effort was required from them to change behaviours. There were no examples of tier 3 services providing advice about behaviour change or self-management strategies, or how people could access such help following surgery. However, this may have been a problem with recall, and that they were offered or received the help but could not remember. The preoperative experiences, expectations and lack of access to behaviour change and self-management services have the potential to impact on postsurgery outcomes.

Commissioning guidance for weight assessment in weight management clinics identifies a lack of evidence on the effectiveness of tier 3 weight management services.^15–16^ While this study does not set out to evaluate tier 3 services, it does highlight the need for such services to prepare people for bariatric surgery by, for example, providing access to behaviour change and self-management strategies, and modifying unrealistic expectations. The variability in tier 3 service provision supports the requirement of commissioning guidance for a structured obesity service pathway to provide opportunities to support people who have spent many years trying to lose and sustain weight loss.

Participants appreciated the fact that services were provided in a non-judgemental manner, but there is potential to expand on current services. Despite being a cost effective treatment, the extent of the success of bariatric surgery relies on the patient’s long-term commitment to behaviour change. Some participants here viewed the surgery as a physical tool to change eating rather than relying on their will power or eating decisions. This suggests naivety regarding postoperative lifestyle change. The potential of positive outcomes following surgery are reduced if patients do not accept the need to modify their eating behaviours.

Unrealistic expectations that have been indicated in this study regarding the perceived level of effort required regarding eating behaviour and weight loss following surgery indicates the need for additional interventions presurgery and postsurgery. Such expectations are understandable if people do not have the opportunity to identify and access the support they require to modify expectations, identify factors that may impede progress, and access support in maintaining healthy eating behaviour. Pfeil *et al*^28^ highlights the additional support that could be provided by bariatric nurses and healthcare professionals in the preoperative stages.

There is the potential to learn from behavioural, self-management interventions in other conditions, for example, the Expert Patient Programme^30^ in long-term conditions, and the Diabetes Education and Self Management for Ongoing and Newly Diagnosed (DESMOND) programme for newly diagnosed type 2 diabetes.^31^ Many such behaviour change interventions are routed in psychological theory and aim to improve psychological wellbeing and illness beliefs, as well as promote behaviour change. These programmes can be cost-effective additions to the management of long-term conditions and help modify illness beliefs. Furthermore, Knutsen and Foss^32^ suggest that mandatory lifestyle courses using empowering education methods may be a powerful approach. This study indicates that such an approach may be appropriate within the bariatric surgery population. The findings here raise the question of whether similar interventions, such as DESMOND, could be developed for people referred for bariatric surgery. Such services could be introduced prior to surgery to prepare people more effectively, but be continued postsurgery to promote sustained self-management and behaviour change. Further research is required to inform the development of such interventions and evaluate their impact on behaviour change, self-management and achieving positive outcomes.^33^

The use of photovoice methodology provided additional insight into the lives of obese people. Participants who engaged with the methods were able to prepare for their interview, considering how their obesity affected their day-to-day lives, and how they expected this to change following their surgery, which added to the richness of the data. Photovoice methodology was a useful way of exploring the experiences of obese people who by the nature of the condition may be a socially isolated and marginalised group of individuals. However, using photovoice techniques in research places an additional demand on participants. The three participants who did not take photographs cited two main reasons which included a lack of time to prepare for the interview and, more specific to obese people, a dislike of having their photographs taken as a result of their obesity.

## Conclusion

This study provides insight into the expectations and experiences of patients in England who have been referred for bariatric surgery. The findings reveal factors that influence their expectations of surgery, and indicate that despite having accessed tier 3 weight management services, these expectations were not always realistic. The study highlights the importance of weight management services assessing and modifying patient's expectations as appropriate. The importance of providing behaviour change and self-management support is also emphasised, and this support needs to take into account the impact of stigmatisation and shame if positive outcomes are to be maximised following surgery. Future research examining postsurgery will be useful to determine the extent to which expectations of the procedure and future life are met, and to develop and evaluate the required interventions.

## References

[1] World Health Organisation. *Physical status: the use and interpretation of anthropometry, report of a WHO expert committee, World Health Organ Tec Rep Ser* 854. Geneva: WHO, 1995. 8594834

[2] Health and Social Care Information Centre. Health Survey for England 2012. London: The Health and Social Care Information Centre, 2013.

[3] World Health Organisation. Obesity: preventing and managing the global epidemic. Report of the WHO Consultation on Obesity. Geneva: WHO, 1998. 11234459

[4] National Institute for Health and Clinical Excellence. Obesity: the prevention, identification, assessment and management of overweight and obesity in adults and children. London: Department of Health, 2006. 22497033

[5] Picot J, Jones J, Colquitt JL The clinical effectiveness and cost-effectiveness of bariatric (weight loss) surgery for obesity: a systematic review and economic evaluation. Health Tech Assess 2009(41) 10.3310/hta13410 19726018

[6] da Silva SS, da Costa Maia A Obesity and treatment meanings in bariatric surgery candidates: a qualitative study. Obes Surg 2012;22:1714–22. 10.1007/s11695-012-0716-y 22820955

[7] Government Office for Science. Tackling obesities: future choices. London: Department for Innovation, 2007.

[8] McKinsey Global Institute. *How the world could better fight obesity*. London: McKinsey & Company, 2014 http://www.mckinsey.com/insights/economic_studies/how_the_world_could_better_fight_obesity (accessed August 2015)

[9] National Institute for Clinical Excellence. Guidance on the use of surgery to aid weight reduction for people with morbid obesity. Technology Appraisal Guidance—No 46. London: Department of Health, 2002.

[10] Gloy VL, Briel M, Bhatt DL Bariatric surgery versus non-surgical treatment for obesity: a systematic review and meta-analysis of randomised controlled trials. BMJ 2013;347:f5934 10.1136/bmj.f5934 24149519 PMC3806364

[11] Chang SH, Stoll CR, Song J The effectiveness and risks of bariatric surgery: an updated systematic review and meta-analysis, 2003–2012. JAMA Surg 2014;149:275–87. 10.1001/jamasurg.2013.3654 24352617 PMC3962512

[12] Torgerson JS, Sjöström L The Swedish Obese Subjects (SOS) study—rational and results. Int J Obes 2001;25:S2–4. 10.1038/sj.ijo.0801687 11466577

[13] NHS Commissioning Board. Clinical Commissioning Policy: Complex and Specialised Obesity Surgery London: NHS Commissioning Board, 2013.

[14] Health and Social Care Information Centre. Statistics on obesity, physical activity and diet. London: The Health and Social Care Information Centre, 2014.

[15] British Obesity & Metabolic Surgery Society. Providing bariatric surgery: BOMMS standards for clinical services & guidance on commissioning. London: BOMMS, 2012 http://www.bomss.org.uk/wp-content/uploads/2014/04/Service_std-2012.pdf

[16] Royal College of Surgeons. Commissioning guide: weight assessment and management clinics (tier 3). London: Royal College of Surgeons, 2014 http://www.rcseng.ac.uk/healthcare-bodies/docs/weight-assessment-and-management-tier-3-services

[17] Ogden J, Clementi C, Aylwin S Exploring the impact of obesity surgery on patients’ health status: a quantitative and qualitative study. Obes Surg 2005;15:266–72. 10.1381/0960892053268291 15802072

[18] Ogden J, Clementi C, Aylwin S The impact of obesity surgery and the paradox of control: a qualitative study. Psychol Heal 2006;21:273 10.1080/14768320500129064 21985121

[19] Wysoker A The lived experience of choosing bariatric surgery to lose weight. J Am Psychiatr Nurs Assoc 2005;11:26 10.1177/1078390305275005

[20] Engstrom M, Wiklund M, Olsen MF The meaning of awaiting bariatric surgery due to morbid obesity. Open J of Nurs 2011;5:1–8. 10.2174/1874434601105010001 PMC3109523 21660178

[21] Bauchowitz A, Azarbad L, Day K Evaluation of expectations and knowledge in bariatric surgery patients. Surg Obes Relat Dis 2007;3:554–8. 10.1016/j.soard.2007.05.005 17702666

[22] Wang C, Burris M Empowerment through photo novella: portraits of participation. Health Educ Q 1994;21:171–86. 10.1177/109019819402100204 8021146

[23] Ritchie J, Lewis J Qualitative research practice: a guide for social science students and researchers. London: Sage, 2003.

[24] Tod AM Interviewing. In: Gerrish K, Lacey EA Research Process in nursing, 6th edn Oxford: Backwells, 2010:345–57.

[25] Coyne IT Sampling in qualitative research. Purposeful and theoretical sampling; merging or clear boundaries? J Adv Nurs 1997;26:623–30. 10.1046/j.1365-2648.1997.t01-25-00999.x 9378886

[26] Baker SE, Edwards R, Doidge M How many qualitative interviews is enough? Expert voices and early career reflections on sampling and cases in qualitative research Southampton: National Centre for Research Methods, 2012.

[27] British Obesity & Metabolic Surgery Society. *Commissioning Guide for Weight Assessment and Management Clinics*. London: BOMSS, 2013.

[28] Pfeil M, Pulford A, Mahon D The patient journey to gastric band surgery: a qualitative exploration. Bariatr Surg Pract Patient Care 2013;8:69–76. 10.1089/bari.2013.9985 24761368 PMC3859175

[29] Steffen KJ, Sarwer DB, Thompson KJ Predictors of satisfaction with excess skin and desire for body contouring surgery after bariatric surgery. Surg Obes Relat Dis 2012;8:92–7. 10.1016/j.soard.2011.06.022 21978749

[30] Richardson G, Kennedy A, Reeves D Cost effectiveness of the Expert Patients Programme (EPP) for patients with chronic conditions. J Epidemiol Community Health 2008;62:361–7. 10.1136/jech.2006.057430 18339831

[31] Khunti K, Gray LJ, Skinner T Effectiveness of a diabetes education and self management programme (DESMOND) for people with newly diagnosed type 2 diabetes mellitus: three year follow-up of a cluster randomised controlled trial in primary care. BMJ 2012;344:e2333 10.1136/bmj.e2333 22539172 PMC3339877

[32] Knutsen IR, Foss C Caught between conduct and free choice—a field study of an empowering programme in lifestyle change for obese patients. Scand J Caring Sci 2011;25:126–33. 10.1111/j.1471-6712.2010.00801.x 20518867

[33] National Institute for Clinical Excellence. Obesity: identification, assessment and management of overweight and obesity in children, young people and adults. Partial update of CG43. London: Department of Health, 2014. 25535639

